# A Rare Case of Incidental Urachal Mucinous Cystadenocarcinoma

**DOI:** 10.7759/cureus.7975

**Published:** 2020-05-05

**Authors:** Jack Komro, Dawood Findakly

**Affiliations:** 1 Internal Medicine, Kirksville College of Osteopathic Medicine, A. T. Still University, Kirksville, USA; 2 Internal Medicine, Creighton University Arizona Health Education Alliance/Valleywise Health Medical Center, Phoenix, USA

**Keywords:** urachus, mucinous cystadenocarcinoma, bladder, ultrasonography, computed tomography, rare cancers

## Abstract

The urachus is a remnant of the embryonic allantois that connects the urinary bladder to the umbilicus. In most cases, it commonly obliterates before birth. Here we present the case of a 60-year-old man with a past medical history of human immunodeficiency virus (HIV) and anal human papillomavirus (HPV). He was referred for an abdominal mass that had been found incidentally while being admitted for an acute kidney injury; it had been excised, and pathological examination showed urachal mucinous cystadenocarcinoma. Surgical excision is performed for the majority of cases with a higher survival rate when diagnosed early.

## Introduction

The urachus is an embryonic allantois duct remnant that attaches the urinary bladder dome to the umbilicus, and it should be obliterated during late-stage gestation. Failure to be obliterated is not uncommon. Thus, if it persists to adult life, a primary urachal cancer (UC) can arise. UC was first described by Hue and Jacquin in 1863. It is rare and typically presents at an advanced stage and has poor outcomes [1]. Hematuria is the most common manifestation in more than 90% of patients, and surgery is the gold standard method for its treatment [2,3]. At the time of diagnosis, approximately 50% of patients have advance stage leading to poor prognosis, and this is due to late presentation [4]. Here we present the case of a 60-year-old man with a past medical history of the human immunodeficiency virus (HIV) and anal human papillomavirus (HPV) who was referred for an abdominal mass that had been found incidentally. He had undergone an elective partial cystectomy, and the subsequent pathological examination showed urachal mucinous cystadenocarcinoma.

## Case presentation

**Figure 1:**
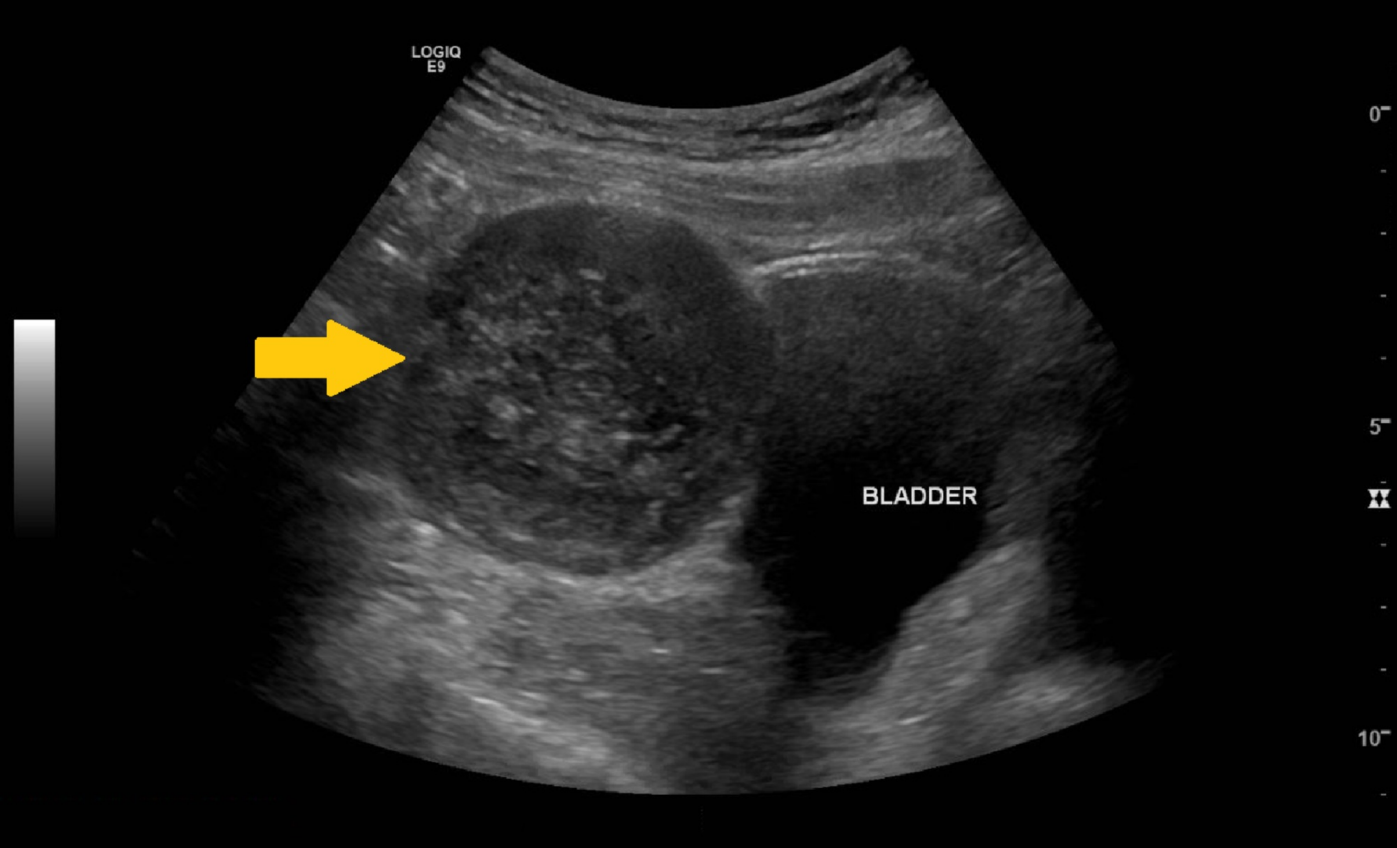
Retroperitoneal ultrasound demonstrating the pelvic mass located just superior to the urinary bladder (yellow arrow).

A 60-year-old man with a past medical history of HIV and anal HPV was referred from his primary care provider to the urology office for an abdominal mass that was found incidentally upon abdominal ultrasound (US) after being admitted to the hospital for an acute kidney injury from dehydration. The patient denied any weight loss, abdominal pain, umbilical discharge, hematuria, or voiding difficulties. He is a former tobacco smoker with a 15-pack-year smoking history and has a history of alcohol abuse. Upon presentation, his vital signs and physical examination were unremarkable. Labs were also unremarkable, and his urinalysis was pertinent for a urine pH of 6 with minimal proteinuria and microscopic hematuria. A retroperitoneal US showed a 7.0-cm midline pelvic mass located just superior to the urinary bladder (Figure 1).

**Figure 2:**
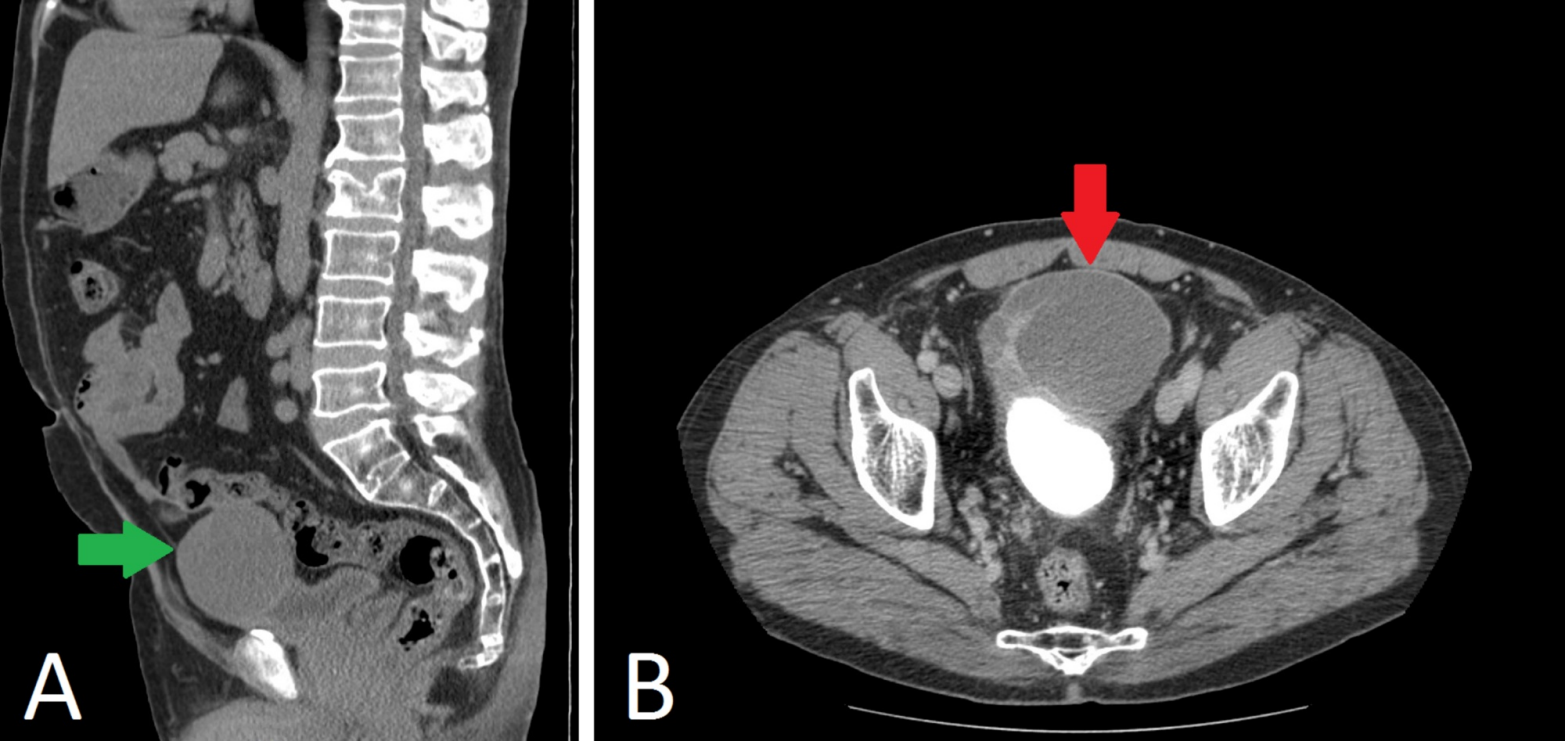
Contrast-enhanced CT scan of the abdomen and pelvis demonstrating the multiseptated cystic mass measuring 6.4 x 7.3 x 7.0 cm in anteroposterior, craniocaudal, and transverse dimensions, which contains multiple thickened, enhancing septations and eccentric wall thickening. This mass is abutting, but not infiltrating the adjacent large bowel wall, and there is a clear fat plane between the anterior aspect of the mass and the inner surface of the lower central abdominal wall. (A) Sagittal plane (green arrow). (B) Axial plane (red arrow).

We performed a subsequent CT scan of the abdomen and pelvis, which demonstrated a 7.3-cm multiseptate anterior bladder wall lesion (Figure 2). 

**Figure 3:**
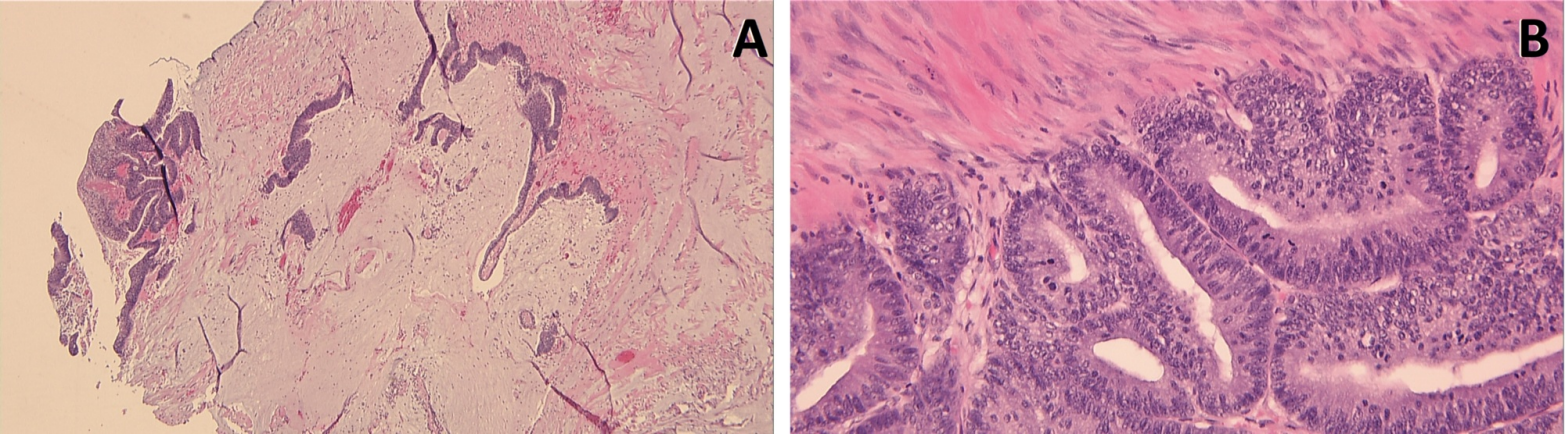
Tumor sections showing urachal carcinoma with abundant mucin filling most of the cystic spaces and occasionally dissecting into the stroma with focal dysplastic epithelium floating the mucin pools, diagnostic of mucinous cystadenocarcinoma. (A) H&E, x40. (B) H&E, x200.

The patient underwent cystoscopy, which ruled out a bladder mass, diverticulum, inflammation, or stones and concluded a mass effect on the anterior bladder wall from a suspected urachal mass with no communication between the mass and the urinary bladder. Eventually, the patient underwent an elective open excision of the lesion which was found to be a sizeable urachal cyst. Because of the adherent nature of the mass to the surrounding tissue, the procedure was combined with partial cystectomy. A pathological examination from the tissue taken from the urachal cyst showed mucinous cystadenocarcinoma favoring urachal origin (Figure 3). The patient was, therefore, diagnosed with Sheldon stage I, Mayo stage I, and T1N0M0 urachal mucinous cystadenocarcinoma. He is doing well and is disease-free at follow-up 10 months after the diagnosis.

## Discussion

Bladder cancer is one of the most common cancers worldwide [5]. UC, however, is exceedingly rare and accounts for less than !% of all bladder cancers with an incidence of one case per million per year [6-8]. UC was first described by Hue and Jacquin in 1863. It is a rare cancer that typically presents at an advanced stage and has poor outcomes [9]. 

The most common demographic is white men over the age of 50 years [6,8,10]. A number of studies have previously described histopathological characteristics. Adenocarcinoma is the most common type of UC, usually having non-cystic and mucinous features [6,8,11]. Given the rarity of UC, relatively few studies have described its characteristics. Increase awareness of it is beneficial to provide a brief overview of the current literature regarding presenting symptoms, diagnosis, staging, and treatment. The most common presenting symptom is macroscopic or microscopic hematuria with multiple less common symptoms such as abdominal pain and mucousuria, among others [10,11]. Cystoscopy, CT, and MRI are the primary diagnostic imaging modalities [10,12]. 

Diagnostic criteria have been modified over time, but the most accepted criteria, according to Hamilou et al., are described by Sheldon et al. in 1984 and Mostofi et al. in 1955 [10,13,14]. The most common location of UC is at the bladder dome. Multiple staging systems are used as the classic TNM staging is not as useful for UC. There is also a higher survival rate with early stages and surgical excision. The role of chemotherapy in the treatment of UC is not well established, although 5-fluorouracil is commonly used. Overall, there is no effective management strategy, and partial cystectomy remains the primary therapeutic option [6,7,10,11]. 

The unique clinical aspect of our patient is the mucinous cystadenocarcinoma diagnosis, a less common UC type with only 10 cases reported in the literature. Our patient was identified early with a Sheldon stage I, Mayo stage I, and T1N0M0, which was discovered incidentally on the abdominal US, treated with open excision of the lesion with partial cystectomy and remains disease-free at a 10-month follow-up. 

## Conclusions

Mucinous cystadenocarcinoma is a rare and aggressive type of UC. Therefore, early diagnosis is crucial in improving patient outcomes. There are no definitive connotative strategies in assessing patients for the presence of UC. Our patient is the 11th case of mucinous cystadenocarcinoma to be published in the literature. This report will help improve our understanding and guide future endeavors in the management of this rare tumor.
